# The Diagnostic Value of Electromyography in Identifying Patients With Pain-Related Temporomandibular Disorders

**DOI:** 10.3389/fneur.2019.00180

**Published:** 2019-03-05

**Authors:** Liliana Szyszka-Sommerfeld, Monika Machoy, Mariusz Lipski, Krzysztof Woźniak

**Affiliations:** ^1^Department of Orthodontics, Pomeranian Medical University, Szczecin, Poland; ^2^Department of Preclinical Conservative Dentistry and Preclinical Endodontics, Pomeranian Medical University, Szczecin, Poland

**Keywords:** orofacial pain, temporomandibular disorders, pain-related temporomandibular disorders, surface electromyography, cleft lip and palate

## Abstract

**Introduction:** Orofacial pain disorders can be divided into several subgroups. One of them is temporomandibular disorders (TMD) with recognizable signs such as joint noises, limitations in the range of motion, or mandibular deviation during function and symptoms—pain in the muscles or joint. Surface electromyography (sEMG) is a diagnostic tool that ensures reliable and valid evaluation of muscle activity. sEMG detects electrical potentials and on this account may conceivably be employed in the TMD recognition. The aim of this study was to assess the sensitivity, specificity, and accuracy of electromyography in diagnosing subjects with temporomandibular disorders, including pain-free TMD and pain-related TMD.

**Methods:** The sample comprised 88 patients with cleft lip and palate and mixed dentition. TMD has been recognized on the grounds of Axis I of the Research Diagnostic Criteria for TMD (RDC/TMD). To evaluate the electrical activity of the temporal and masseter muscles in the rest position and during maximum voluntary contraction, a DAB-Bluetooth Instrument (Zebris Medical GmbH, Germany) was used. The analysis of the receiver operating characteristic (ROC) curve gave information about accuracy, cut-off point value, sensitivity and specificity of the normalized sEMG data.

**Results:** The highest diagnostic efficiency of sEMG in terms of identifying subjects with TMD and pain-related TMD was observed for the mean values of temporal and masseter muscle activity as well as the Asymmetry Index of the masseter muscles in a rest position. A moderate degree of EMG accuracy in differentiating between pain-related TMD and non-TMD children was observed for the mean values of masseter muscle activity and the Asymmetry Index of the masseter muscles at rest.

**Conclusion:** An evaluation of electromyography exhibits its diagnostic usability in recognition of patients with pain-related TMD and it could be used as an adjunctive tool in the identification of this disorder.

**Clinical Trial Registration:** This clinical research was registered in the ClinicalTrials.gov database under the number NCT03308266.

## Introduction

Orofacial pain disorders can be divided into several subgroups. One of them are temporomandibular disorders (TMD) with recognizable signs such as joint noises, limitations in the range of motion, or mandibular deviation during function and symptoms—pain in the muscles or joint (1, 2). The multifactorial etiology of this condition hinders the precise diagnosis and requires many tools and activities to draw correct conclusion (3–6). An accurate medical history and standardized tests and examinations are considered to be the standard reference point. In clinical evaluations of many TMD cases to provide valid quantitative data it is advisable to collect additional information by using electronic devices (6–11).

The one of the most current and useful tool for TMD assessment are the Research Diagnostic Criteria for Temporomandibular Disorders (RDC/TMD) (2, 12). The criteria provide a holistic approach to the TMD identification by diagnosing both physical and psychosocial aspects and therefore ensuring standardized procedures for epidemiological studies, and a comparison with the results of other similar studies (2, 13, 14). An accurate recognition of TMD is especially important in the case of children, as early identification of TMD in childhood could be useful when minimizing the risk of developing chronic pain and preventing persistent or severe TMD problems during adolescence (15). Children with congenital abnormalities, such as cleft lip and palate (CLP) are potentially at risk of developing TMD due to psychosocial burdens, as well as malocclusions predisposing them to this condition (16, 17). The signs and symptoms of TMD occur more frequently in children with CLP than in children and adolescents in the general population (15–21).

One of the only diagnostic tool that allows an evaluation of muscle function and efficiency by directly and objectively detecting their electrical potentials is electromyography (22). This method has been widely used for the diagnosis of patients with general muscle disorders, neuromuscular diseases or diseases affecting neuromuscular performance (23, 24). Surface electromyography (sEMG) as global electromyography, in contrary to the quantitative intramuscular electromyography that uses intramuscular needle electrodes, “uses surface electrodes and detects superimposed motor unit action potentials from many fibers, as opposed to the single ones recorded by the intramuscular type” (23). Wherefore the analysis of the sEMG findings is limited to three main subjects: “general muscle activity, the cooperation of different muscles, and the variability of their activity over time” (23). The most important advantage of sEMG is its non-invasiveness (23). It is a painless and innocuous method for evaluating muscle function that may conceivably be used in the TMD identification (6, 25). Nevertheless, its application in the recognition of this disorder remains disputable due to significant variability in the results described in the literature (6, 26). A systematic review gained no attestation to support the efficacy of surface electromyography as a diagnostic tool for TMD (2, 27). On the other hand, a more recent study presented the moderate accuracy of sEMG values for masticatory muscles when assessing TMD in adults (6).

The most dominant TMD conditions are pain-related temporomandibular disorders (TMD-P) (28). The primary manifestation of TMD-P is a persistent, recurring, or chronic pain that affects jaw muscles, the temporomandibular joint (TMJ), and/or adjacent structures (13, 29). Subjects diagnosed with TMD-P modify a tension of their masticatory muscles. Pain induces adaptations by reworking muscle activity in order to shield the masticatory motor system from possible trauma (30, 31). During muscle contraction pain can cause greater alteration in electromyographical activity, which in turn may affect the accuracy of this equipment (6, 32).

As the assessment of subjects with TMD by using the sEMG remains disputable and there have been no previous studies assessing the efficacy of EMG in diagnosing TMD, including pain-related TMD in CLP children, it is important that we undertake research in this field. Electromyographic study of masticatory muscle activity in cleft lip and palate subjects with a TMD-pain diagnosis have previously been completed (15).

The aim of the present study was to assess the sensitivity, specificity, and accuracy of electromyography in diagnosing TMD, including both pain-free TMD (TMD-PF) and pain-related TMD (TMD-P) in cleft lip and palate patients. We hypothesized the diagnostic inefficiency of electromyography in identifying CLP patients with TMD.

## Methods

This clinical research was registered in the ClinicalTrials.gov database as number NCT03308266. The protocol was approved by the Local Bioethics Committee of the Pomeranian Medical University (number KB-0012/08/15). The children's parents were notified about the test procedures and gave written informed consent to all the performers' procedures in accordance with the Declaration of Helsinki.

The sample comprised 88 patients with cleft lip and palate and mixed dentition. Following an evaluation based on algorithms for Axis I of the Research Diagnostic Criteria for Temporomandibular Disorders (RDC/TMD) (13), children diagnosed with pain-free TMD were included in the non-pain TMD group (Group 1), subjects diagnosed with pain-related TMD were included in the TMD-pain group (Group 2) and patients with no TMD diagnosis comprised the non-TMD group (Group 3). Participants were selected from the group of 100 patients who had been referred to Orthodontic Cleft Care Center in Poland. After adaptation of the exclusion criteria there were 88 patients qualified for further examination left. Group 1 comprised 25 children (12 girls and 13 boys) with a mean age of 9.4 ± 1.7 with CLP and a pain-free TMD diagnosis. Group 2 included 30 CLP subjects (14 girls and 16 boys) with a mean age of 9.1 ± 1.5 with a TMD-P diagnosis. Group 3 comprised 33 CLP subjects (16 girls and 17 boys) with a mean age of 8.9 ± 1.5 with no TMD. The exclusion criteria for all groups included the following: the presence of a cleft lip and palate with other congenital abnormalities, the presence of systemic or rheumatologic diseases, a history of cervical spine or TMJ surgery, trauma or deformities, as well as completed orthodontic or masticatory motor system dysfunction treatment.

The function of the masticatory motor system was evaluated by taking into consideration a clinical and electromyographic the analysis. The general medical histories of the patients were taken, which provided information on the patients' masticatory motor systems, including subjective TMD symptoms, such as jaw pain during function, frequent headaches, jaw stiffness/fatigue, difficulty of mouth opening in normal plane, teeth gnashing, and TMJ sounds (33). Axis I scoring of the Research Diagnostic Criteria for TMD (RDC/TMD) (13) was used to assess children for the presence of temporomandibular disorders with the same trained examiner. The RDC/TMD was used as the gold standard. The temporomandibular disorder was recognized when the clinical signs fulfilled the criteria of RDC/TMD such as “pain on palpation, mandibular range of motion, associated pain (jaw opening pattern, unassisted opening, maximum assisted opening, mandibular excursive and protrusive movements), sounds coming from the TMJ, and tenderness induced by muscle and joint palpation” (34).

To take electromyographical recordings a DAB-Bluetooth Instrument (Zebris Medical GmbH, Germany) was used by a single experienced researcher. We followed the methods previously described by Szyszka-Sommerfeld et al. (15, 22). In the process of recordings, the head was unsupported, in natural head position (NHP) (35, 36). The masseter and temporal anterior muscles were examined with disposable silver/silver chloride (Ag/AgCl), self-adhesive, bipolar surface electrodes at an interelectrode distance of 20 mm (Noraxon Dual Electrode, Noraxon, USA) positioned on the muscle bellies parallel to the muscle fibers (“temporal anterior: vertically along the anterior muscular margin, around the coronal suture; masseter: parallel to the muscle fibers, with the upper pole of the electrode at the intersection between the tragus-labial commissure and the exocanthion-gonion lines”) (37, 38). Reference electrodes were applied in positions “inferior and posterior to the right ear” (39).

The main hindrance of the examination could be the skin impedance. The obstacle was withdrawn by cleaning the skin surface with 70% ethyl alcohol and dried prior to the placement of the electrode (39). The proper preparation was proven by carrying an impedance test that was performed with Metex P-10 a measuring device (Metex Instruments Corporation, Korea). If the test produced a positive result showing low skin tissue impedance, further examinations would be conducted (22). The EMG assessments were performed 5 min later. EMG activity was then recorded during three different tasks, in the same way as was previously described by Szyszka-Sommerfeld et al. (22):

1. Rest activity of the masticatory muscles was performed “in the clinical rest position.”
2. Maximum voluntary clench (MVC)—was performed “in the intercuspal position and the subject was asked to clench as hard as possible for 5 s.”
3. Maximum voluntary clench (MVC)—was performed “with two 10-mm thick cotton rolls positioned on the mandibular second premolars and molars, or on the mandibular second milk molars and the first permanent molars and the subject was asked to clench as hard as possible for 5 s.”

The movements were repeated at least three times to ascertain stability. Between each of every recording 5 min of rest was granted a permission to avoid any effects of fatigue.

The EMG signals after the registration were processed by amplification, digitization and digital filtration. The DAB-Bluetooth Instrument was ported to a computer, which enabled the data graphical presentation and further quantitative and qualitative analyses. The analysis encompassed mainly the normalization process as the essential procedure for the initial processing of raw data to ensure reliable further analysis. The EMG recordings ought to be mutually likened to the electrical muscle activity detected during certain standardization recordings, such as MVC. The electrical potentials collected in maximum voluntary clenching are reported to have the highest repeatability. Amidst the various protocols, MVC on cotton rolls is reported to vary inter-individually in the smallest extent and on that account a method based on this standardization is now regularly used (37, 40, 41). On the grounds, normalization included referring the raw results (the mean values of the electrical potentials) to the data acquired from each patient after clenching on cotton rolls (reference values) in accordance with the following formula: “mean values (μV) during rest position or MVC / mean values (μV) during MVC with two 10-mm cotton rolls × 100%” (22). EMG potentials of every analyzed muscle were submitted as a percentage of the maximum voluntary clenching value with cotton rolls (unit μV/μV%). Regularly, normalized EMG data will implement information about the impact of “teeth contact on neuromuscular activity, while avoiding individual variability (anatomical variations, physiological and psychological status, etc.) and technical variations (muscle cross-talk, electrode position, skin, and electrode impedance, etc.)” (15, 41).

Finally, the Asymmetry Index (As, unit %) was recorded to assess asymmetry concerning the activity of the left and right masticatory muscles using the following formula:

$$\begin{array}{l} \text{As}=\overset{N}{\underset{i=1}{\sum }}|R_{i}-L_{i}|/\overset{N}{\underset{i=1}{\sum }}\left(R_{i}+L_{i}\right)\times 100\; \end{array}$$

This ranges from 0% (total symmetry) to 100% (total asymmetry) (42).

In order to achieve a proper statistical result, the Levene test was used to evaluate homogeneity of variance and the Kolmogorov-Smirnov test was used to assess normality. To verify the research hypotheses toward the presence or absence of differences between the mean values of the independent variables the Student *t*-test and the Mann-Whitney *U* test were applied. The level of significance was set at *p* = 0.05. The area under the curve (AUC) was determined by the receiver operating characteristic (ROC) curve. It gave information about accuracy, cut-off point value, sensitivity (Se) and specificity (Sp) of the normalized sEMG data. The classification of the AUC was as follows: “0.5, result due to chance; >0.5–0.7, low accuracy; >0.7–0.9, moderate accuracy; >0.9–<1.0, high accuracy; and 1.0, a perfect test” (6, 43, 44).

## Results

Table 1 shows the diagnostic efficiency of EMG in identifying CLP subjects with TMD (pain-free TMD and pain-related TMD; Group 1 and Group 2 vs. Group 3). The analysis of the ROC curve demonstrated that the diagnostic efficiency of electromyography in distinguishing between TMD and non-TMD children was highest in the case of estimators of distribution of variables, such as the mean values of temporal and masseter muscle activity in a rest position (temporal: AUC = 0.664, the standard error of mean [SEM] = 0.058, *p* = 0.0110; cut-off point = 6.45 μV/μV%, Se = 63%, 1–Sp = 38%; masseter: AUC = 0.662, SEM = 0.062, *p* = 0.0120, cut-off point = 3.80 μV/μV%, Se = 77%, 1–Sp = 50%), as well as the Asymmetry Index for the masseter muscles at rest (AUC = 0.647, SEM = 0.063, *p* = 0.0220, cut-off point = 4.47%, Se = 48%, 1–Sp = 22%).

**Table 1:** Data of the area under ROC curve, best cut-off value, sensitivity and specificity of EMG in identifying children with TMD (Group 1 and 2) and non-TMD subjects (Group 3).

**Activity**	**Region**	**Variable**	**AUC (95% CI)**	**SEM**	***P*-value**	**Cut-off value**	**Se [%]**	**1–Sp [%]**
Rest	TA	EA [μV/μV%]	0.664 (0.550–0.777)	0.058	0.0110^*^	6.45	63.0	38.0
		As [%]	0.616 (0.485–0.746)	0.067	0.0726	18.43	86.0	56.0
	MM	EA [μV/μV%]	0.662 (0.540–0.783)	0.062	0.0120^*^	3.80	77.0	50.0
		As [%]	0.647 (0.524–0.770)	0.063	0.0220^*^	4.74	48.0	22.0
MVC	TA	EA [μV/μV%]	0.595 (0.474–0.717)	0.062	0.1380	103.89	52.0	31.0
		As [%]	0.552 (0.431–0.673)	0.062	0.4198	9.38	41.0	25.0
	MM	EA [μV/μV%]	0.537 (0.413–0.660)	0.063	0.5699	77.94	32.0	16.0
		As [%]	0.612 (0.489–0.736)	0.063	0.0805	6.32	59.0	34.0

MVC, maximum voluntary contraction; TA, anterior temporal muscle; MM, masseter muscle; EA, electrical activity; As, asymmetry index; AUC, area under ROC curve; SEM, standard error of mean; Se, sensitivity; Sp, specificity;

* *Statistically significant difference*.

Table 2 presents the diagnostic value of EMG in identifying CLP children with pain-related TMD (Group 2 vs. Group 3). The highest diagnostic efficiency of EMG in discriminating between TMD-P and non-TMD subjects was observed for the mean values of temporal and masseter muscle rest activity (for temporal muscle AUC = 0.655, SEM = 0.069, *p* = 0.0342, cut-off point = 6.45 μV/μV%, Se = 68%, 1–Sp = 38%; for masseter muscle: AUC = 0.728, SEM = 0.064, *p* = 0.0018, cut-off point = 3.80 μV/μV%, Se = 90%, 1–Sp = 50%), as well as the Asymmetry Index for the masseter muscles at rest (AUC = 0.723, SEM = 0.064, p = 0.024, cut-off point = 10.12%, Se = 87%, 1–Sp = 50%). A moderate degree of EMG accuracy in terms of differentiating between TMD-P and non-TMD children was observed for the mean values of masseter muscle activity and the Asymmetry Index of the masseter muscles at rest position (Table 2).

**Table 2:** Data of the area under ROC curve, best cut-off value, sensitivity and specificity of EMG in identifying children with TMD-P (Group 2) and non-TMD subjects (Group 3).

**Activity**	**Region**	**Variable**	**AUC (95% CI)**	**SEM**	***P*-value**	**Cut-off value**	**Se [%]**	**1–Sp [%]**
Rest	TA	EA [μV/μV%]	0.655 (0.521–0.790)	0.069	0.0342^*^	6.45	68.0	38.0
		As [%]	0.620 (0.468–0.705)	0.073	0.1018	17.57	94.0	56.0
	MM	EA [μV/μV%]	0.728 (0.604–0.853)	0.064	0.0018^*^	3.80	90.0	50.0
		As [%]	0.723 (0.597–0.848)	0.064	0.0024^*^	10.12	87.0	50.0
MVC	TA	EA [μV/μV%]	0.607 (0.466–0.748)	0.072	0.1450	109.58	65.0	41.0
		As [%]	0.580 (0.437–0.722)	0.073	0.2774	8.97	48.0	28.0
	MM	EA [μV/μV%]	0.593 (0.451–0.734)	0.072	0.2059	104.63	71.0	47.0
		As [%]	0.630 (0.489–0.771)	0.072	0.0761	6.59	65.0	34.0

MVC, maximum voluntary contraction; TA, anterior temporal muscle; MM, masseter muscle; EA, electrical activity; As, asymmetry index; AUC, area under ROC curve; SEM, standard error of mean; Se, sensitivity; Sp, specificity;

* *Statistically significant difference*.

The results showed that the highest diagnostic efficiency of EMG in identifying pain-free TMD children (Group 1 vs. Group 3) was achieved in the case of the mean values of temporal muscle activity in the mandibular rest position (AUC = 0.658, SEM = 0.076, *p* = 0.0427, cut-off point = 7.43 μV/μV%, Se = 52%, 1–Sp = 19%, Table 3).

**Table 3:** Data of the area under ROC curve, best cut-off value, sensitivity and specificity of EMG in identifying children with TMD-PF (Group 1) and non-TMD subjects (Group 3).

**Activity**	**Region**	**Variable**	**AUC (95% CI)**	**SEM**	***P*-value**	**Cut-off value**	**Se [%]**	**1–Sp [%]**
Rest	TA	EA [μV/μV%]	0.658 (0.509–0.806)	0.076	0.0427^*^	7.43	52.0	19.0
		As [%]	0.610 (0.463–0.757)	0.075	0.1570	22.16	92.0	66.0
	MM	EA [μV/μV%]	0.556 (0.400–0.711)	0.079	0.4742	5.00	52.0	28.0
		As [%]	0.554 (0.403–0.705)	0.077	0.4892	4.68	40.0	22.0
MVC	TA	EA [μV/μV%]	0.535 (0.380–0.690)	0.079	0.6525	113.88	59.0	40.0
		As [%]	0.518 (0.360–0.675)	0.081	0.8219	13.11	28.0	13.0
	MM	EA [μV/μV%]	0.533 (0.373–0.693)	0.082	0.6700	119.31	78.0	52.0
		As [%]	0.591 (0.441–0.740)	0.076	0.2436	4.12	76.0	50.0

MVC, maximum voluntary contraction; TA, anterior temporal muscle; MM, masseter muscle; EA, electrical activity; As, asymmetry index; AUC, area under ROC curve; SEM, standard error of mean; Se, sensitivity; Sp, specificity;

* *Statistically significant difference*.

The efficiency of the normalized EMG data for all variables during rest and MVC was higher in assessments of TMD-P than in diagnoses of TMD and TMD-PF subjects (Tables 1–3).

## Discussion

In this research we evaluated the diagnostic value of surface electromyography as a technique for identifying temporomandibular disorders in cleft lip and palate children. The non-pain TMD and pain-related TMD groups were compared with a control group with no TMD. An analysis of the results demonstrated that the highest diagnostic efficiency for EMG in identifying subjects with TMD (TMD-PF and TMD-P) and patients with TMD-P was achieved for such variables as the mean values of temporal and masseter muscle activity and the Asymmetry Index of the masseter muscles in the rest position, as well as for the mean values of temporal muscle rest activity to diagnose children with pain-free TMD. A moderate degree of EMG accuracy in differentiating between pain-related TMD and no TMD patients was observed in the case of the mean masseter muscle activity values and the Asymmetry Index of the masseter muscles at rest position.

As mentioned earlier, surface electromyography (sEMG) by dint of detecting electrical potentials is the most reliable and valid method for assessing muscle function and efficiency (39). The EMG method is harmless, painless and innocuous which is an utmost importance when conducting studies involving children (22, 23). In our study there were no difficulties with reference to the cooperation of the children during EMG recordings. The diagnostic value of EMG in identifying pain-related TMD in children, has yet to be agreed in the literature. The present study provides the first ever data on the accuracy, sensitivity and specificity of normalized sEMG values in the recognition of pain-related TMD in CLP children in the rest position and during MVC. The data analysis encompassed mainly the normalization process. This scheme was essential for the initial processing of raw data to ensure interindividual comparisons (22, 37). EMG potentials of every analyzed muscle were submitted as a percentage of the MVC value using cotton rolls. In order for the research to be objective, any variability arising from skin and electrode impedance, electrode positioning or relative muscular hypo- or hypertrophy should be obviated (37–46).

The previous study concerning masticatory muscle EMG activity in CLP children diagnosed with TMD-P based on the RDC/TMD criteria confirmed that in comparison to non-TMD patients subjects diagnosed with pain-related TMD have altered temporal and masseter muscle activity. It was noted that altered muscle electrical activity in subjects with TMD-P can affect muscle fatigue, and can, as a consequence, have an impact on every function they perform in the stomatognathic system (15).

The diagnostic effectiveness of selected non-invasive methods of instrumental diagnostics to identify temporomandibular disorders have previously been discussed. The authors demonstrated considerable variability in the diagnosis of this disorder (32, 47–49). The assessment of patients with TMD by using the sEMG remains disputable. The diagnostic accurateness of surface electromyography and kinesiography devices in the diagnosis of individuals with myofascial pain of masticatory muscles was assessed by Manfredini et al. (32). The authors reported an unacceptable efficiency of sEMG at rest in discriminating between myogenous TMD-pain and asymptomatic subjects (AUC = 0.28–0.48) and fair to excellent degree of EMG accuracy during clenching tasks (AUC > 0.7). It has been also promulgated and should be emphasized that the use of EMG indices in the diagnosis of myogenous TMD should be used attentively, rarely if ever, due to the potential risk of false-positive results (6, 32). Contrarily, De Felício et al. (50) remarked a positive correlation between sEMG indices and TMD-signs and symptoms, implying potential sEMG efficiency in distinguishing between myogenous TMD plus disc displacement with reduction and normal subjects. Castroflorio et al. (51) and Lauriti et al. (9) found that sEMG indices of the masticatory muscles are reproducible in identifying TMD and non-TMD patients at rest position and during MVC on parafilm (9).

The efficacy of the sEMG in identifying TMD patients was also confirmed by Woźniak (52). It has been stated that the most significant recordings were those of temporal muscles in maximum voluntary clenching (AUC = 0.918) and changes in the mean power frequency (MPF%) of the masseter muscle during a 10-s maximum voluntary clenching in an intercuspal position (AUC = 0.911).

Santana-Mora et al. (53) determined the diagnostic value of EMG in distinguishing between TMD and non-TMD patients. They reported a moderate effectiveness of sEMG at rest position in discriminating between patients without TMD and those with TMD (Se = 0.547, Sp = 0.842) only in the left temporal muscle (AUC = 0.660). Glaros et al. (54) observed the diagnostic efficiency for EMG in differentiating between the TMD and control groups (Se = 68.5, Sp = 66.8), specifically in the case of the left anterior temporal and left masseter muscles.

A study performed by Berni et al. (6) confirmed the findings of Santana-Mora et al. (53) and Glaros et al. (54). Berni et al. (6) analyzed the accuracy, sensitivity and specificity of the anterior temporal, masseter and suprahyoid EMG muscle activity in the diagnosis of myogenous TMD in women at rest position and during maximum voluntary clenching on parafilm. In all examined muscles, with reference to the diagnosis of TMD at rest and in the suprahyoid muscles during MVC on parafilm, a moderate degree of sEMG accuracy was detected (AUC = 0.747–0.848, Se = 71.3–80%, Sp = 60.5–76.6%). Contrarily, the authors observed unacceptable degrees of accuracy for masseter and temporal muscles during maximum voluntary clenching with parafilm (AUC < 0.5). It was confirmed that the sEMG is an auxiliary tool in the identification of the myogenous TMD.

Our study also corroborates these results, since a moderate degree of EMG accuracy was observed for masseter muscle rest activity and the Asymmetry Index for the masseter muscles at rest and low accuracy for all variables during MVC in differentiating between pain-related TMD and non-TMD patients. These findings suggested that EMG studies could be used as an adjunctive tool in evaluating of this disorder.

The study limitations were as follows: the relatively small number of subjects involved, along with a heterogenous group due to comparatively wide age range of patients. Hence some differences between individuals may result from variations in neuromuscular system development which can vary according to the age. It should also be noted that the TMD groups included both joint- and muscle-related disorders, while EMG activity may vary in these subgroups of patients. On that account, a further study would be necessary to substantiate the study results.

## Conclusions

The findings and limitations of this study lead to the conclusion that an evaluation of electromyography is diagnostically useful in identifying patients with pain-related TMD. It could be used as an auxiliary and additional tool in the recognition of this disorder. Most essential in this regard were the EMG recordings of the masseter muscle rest activity and the Asymmetry Index for the masseter muscles in the rest position.

## Data Availability

All datasets generated for this study are included in the manuscript and/or the supplementary files.

## Author Contributions

LS-S prepared the conception and design of the study. LS-S and KW collected the data. LS-S analyzed the data. ML performed the statistical analysis. LS-S and MM prepared the manuscript. All authors revised, read, and approved the submitted version.

### Conflict of Interest Statement

The authors declare that the research was conducted in the absence of any commercial or financial relationships that could be construed as a potential conflict of interest.
